# Effect of a Biobehavioral Environmental Approach on Sleep in Low-Income Older Adults

**DOI:** 10.1093/geroni/igab046.130

**Published:** 2021-12-17

**Authors:** Junxin Li, Safiyyah Okoye, Lena Sciarratta, Sarah Szanton

**Affiliations:** 1 Johns Hopkins University, Baltimore, Maryland, United States; 2 Johns Hopkins School of Nursing, Baltimore, Maryland, United States

## Abstract

Low socioeconomic status and disability are independent risk factors for disturbed sleep. The CAPABLE intervention used a multidisciplinary team approach of occupational therapist, nurse, and handyworker to reduce functional disability in low-income older adults. The 6-month intervention may benefit sleep as the intervention addressed multiple individual factors associated with sleep quality, including pain, depression, communication, mobility, strength, and balance. This study examined the effect of the CAPABLE intervention on actigraphy-measured sleep in a sub-sample of 73 older adults from the CAPABLE trial (26 intervention vs. 47 control). The sample was aged 75.8±7.45 years, 86.3% female, and 84.9% African American. No significant group differences in sleep parameters were found at 6-month, controlling for baseline values. The intervention resulted in a 5.56% increase in sleep efficiency (95% CI= [1.39, 9.71], Cohen’s d=0.54), and 7.39 minutes decrease in sleep onset latency (95% CI= [0.10, 14.5], Cohen’s d=0.41) within the intervention group at 6-months.

